# KFuji RGB-DS database: Fuji apple multi-modal images for fruit detection with color, depth and range-corrected IR data

**DOI:** 10.1016/j.dib.2019.104289

**Published:** 2019-07-19

**Authors:** Jordi Gené-Mola, Verónica Vilaplana, Joan R. Rosell-Polo, Josep-Ramon Morros, Javier Ruiz-Hidalgo, Eduard Gregorio

**Affiliations:** a Research Group in AgroICT & Precision Agriculture, Department of Agricultural and Forest Engineering, Universitat de Lleida (UdL) – Agrotecnio Center, Lleida, Catalonia, Spain; b Department of Signal Theory and Communications, Universitat Politècnica de Catalunya, Barcelona, Catalonia, Spain

**Keywords:** Multi-modal dataset, Fruit detection, Depth cameras, RGB-D, Fruit reflectance, Fuji apple

## Abstract

This article contains data related to the research article entitle “Multi-modal Deep Learning for Fruit Detection Using RGB-D Cameras and their Radiometric Capabilities” [1]. The development of reliable fruit detection and localization systems is essential for future sustainable agronomic management of high-value crops. RGB-D sensors have shown potential for fruit detection and localization since they provide 3D information with color data. However, the lack of substantial datasets is a barrier for exploiting the use of these sensors. This article presents the KFuji RGB-DS database which is composed by 967 multi-modal images of Fuji apples on trees captured using Microsoft Kinect v2 (Microsoft, Redmond, WA, USA). Each image contains information from 3 different modalities: color (RGB), depth (D) and range corrected IR intensity (S). Ground truth fruit locations were manually annotated, labeling a total of 12,839 apples in all the dataset. The current dataset is publicly available at http://www.grap.udl.cat/publicacions/datasets.html.

**Table:** Specifications table

Subject area	*Machine learning, computer vision, deep learning, agronomy*
More specific subject area	*Image fusion, Precision agriculture.*
Type of data	*Multi-modal images with color (RGB), depth (D), and range-corrected IR intensity (S).*
How data was acquired	*The images were acquired using Microsoft Kinect v2.*
Data format	*Raw images: JPG* *Raw point clouds: MAT* *Pre-processed images: JPG (color channels) and MAT (depth and range-corrected IR channels)* *Annotations: CSV and XLM.*
Experimental factors	*Different image modalities have been registered to have pixel-wise correspondence between image channels.*
Experimental features	*All captures were carried out during the night, using artificial lighting.*
Data source location	*Data were acquired in Tarassó Farm, a commercial apple field located in Agramunt, Catalonia, Spain (E:* 336297 m N*: 4623494 m 31 N 312 m a.s.l., UTM31T - ETRS89).*
Data accessibility	http://www.grap.udl.cat/publicacions/datasets.html
Related research article	*Gené-Mola J, Vilaplana V, Rosell-Polo J.R, Morros J.R, Ruiz-Hidalgo J, Gregorio E. Multi-modal Deep Learning for Fruit Detections Using RGB-D Cameras and their Radiometric Capabilites. Computers and Electronics in Agriculture (2018) 162, 689–698.*https://doi.org/10.1016/j.compag.2019.05.016[1]

**Table:** 

**Value of the data** • First dataset for fruit detection that contains 3 different modalities: color, depth and range corrected IR intensity. • The presented dataset could be used in the development and training of fruit detection systems with applications in yield prediction, yield mapping and automated harvesting. • Compilation of this database allows fusing RGB-D and radiometric information obtained with Kinect v2 for fruit detection.

## 1 Data

**Fig. 1:**
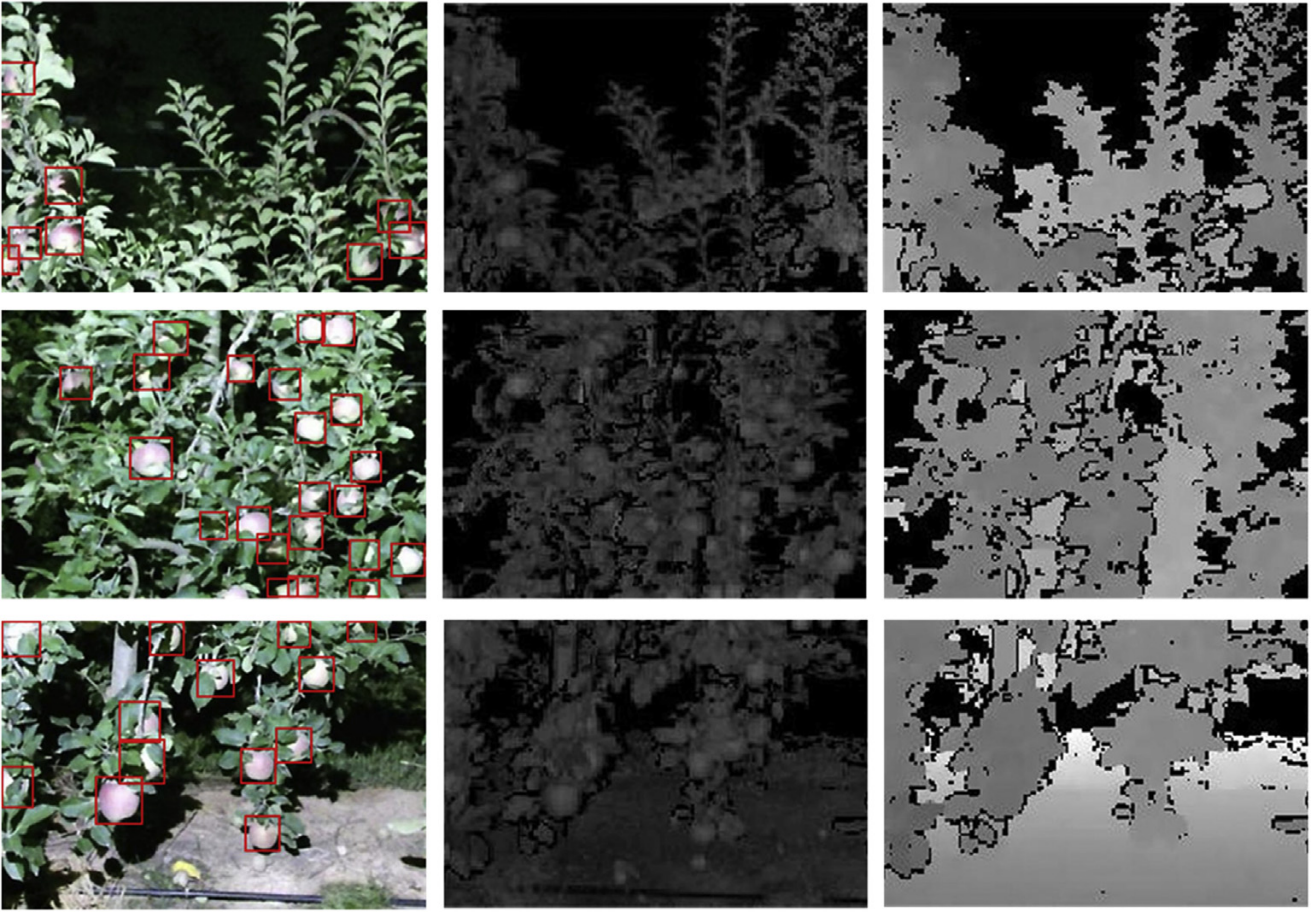
Selection of 3 multi-modal images and the corresponding ground truth fruit locations (red bounding boxes). Each image column corresponds to a different image modality: RGB, S and D, respectively.

The KFuji RGB-DS database contains a total of 967 multi-modal images of Fuji apples on trees and the corresponding ground truth fruit location annotations. Each image contains data from three different modalities: color (RGB), depth (D), and range-corrected IR intensity (S). Fig. 1 illustrates three selected images from de dataset, showing ground truth annotations and the modalities that composes each image.

This dataset was built to be used for training, validation and benchmarking of fruit detection algorithms using RGB-D sensors. For instance, in Ref. [1], the deep convolutional neural network Faster R-CNN [2] was used to detect and localize fruits from the presented dataset.

Images are 548 × 373px and were saved in three different files:

• RGB_hr_ (high resolution color image): Raw color image. These images are saved in 8-bit JPG files.
• RGB_p_ (projected color image): Projection of the color 3D point cloud onto the camera focal plane. The RGB_p_ and the D-S modalities are obtained following the same procedure, allowing the comparison between these modalities for fruit detection. These images are saved in 8-bit JPG files.
• DS (depth and range-corrected IR image): Projection of the range-corrected IR 3D point cloud onto the camera focal plane. The D channel corresponds to the depth values, while the S channel corresponds to the range-corrected IR intensity values. These modalities are saved in a unique 64-bit MAT file.

S and D data were normalized between 0 and 255 –like RGB images-to achieve similar mean and variance between channels. This normalization allows a faster learning convergence of machine learning algorithms (such as deep convolutional neural networks).

All images were manually annotated with rectangular bounding boxes, labelling a total of 12,839 apples in all the dataset. Annotations are provided in XLM and CSV formats, where each row corresponds to an apple annotation, giving the following information: item, topleft-x, topleft-y, width, height, label id.

## 2 Experimental design, materials, and methods

The data acquisition was carried out in a commercial Fuji apple orchard (Malus domestica Borkh. cv. Fuji), three weeks before harvesting (85 BBCH growth stage [3]). The RGB-D sensors used were two Microsoft Kinect v2 (Microsoft, Redmond, WA, USA), which are composed by an RGB camera and a time-of-flight (ToF) depth sensor. For each capture, the sensor provides a 3D point cloud with RGB and backscattered IR intensity data, and a raw RGB image. Due to the performance of the depth sensor drops under direct sunlight exposure [4], data was acquired at night using artificial lighting.

**Fig. 2:**
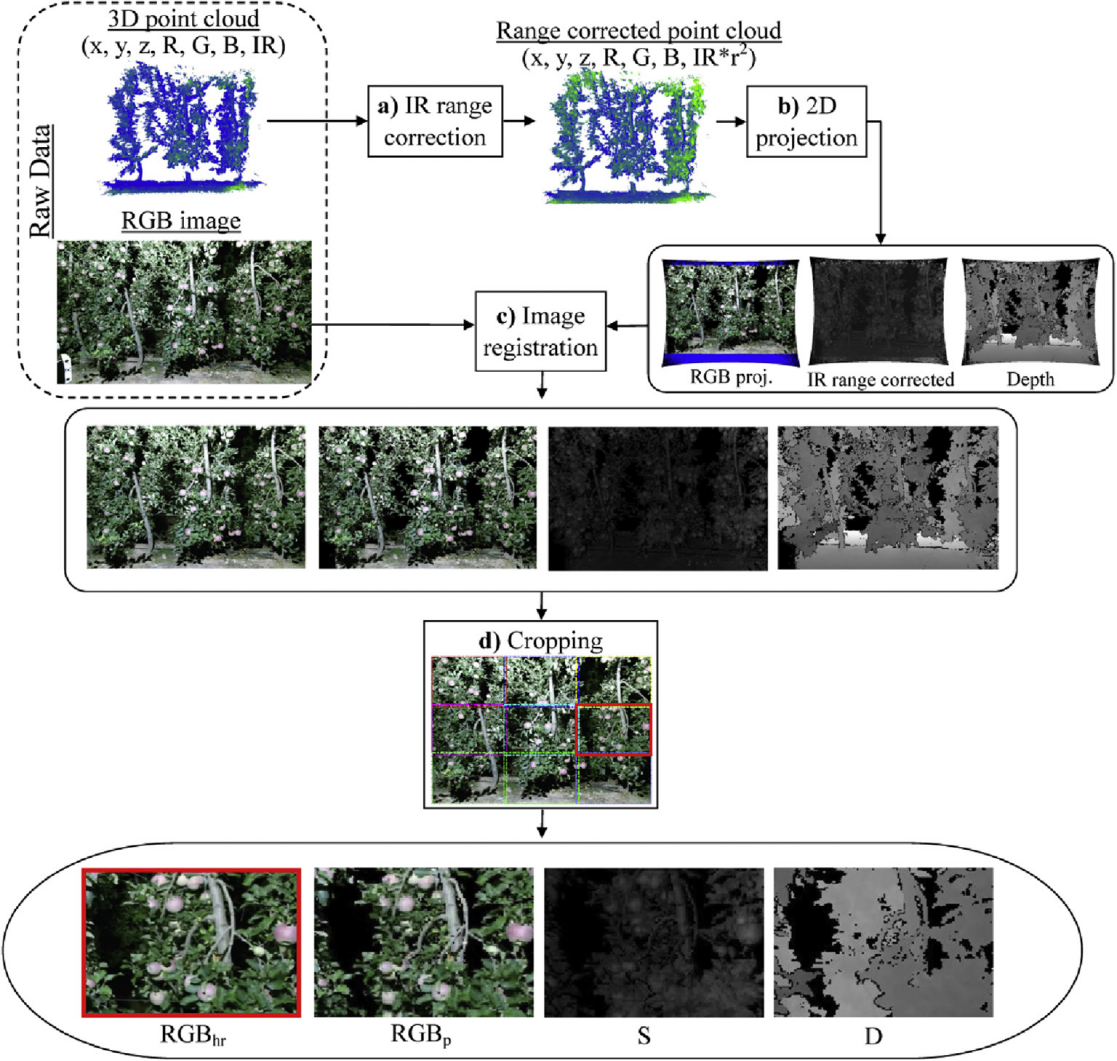
Data preparation outline.

Pre-processing of data was carried out to build the multi-modal images with pixel-wise correspondence between channels. Fig. 2 shows an outline of the data preparation steps. To overcome the IR signal attenuation, the IR intensity data was range-corrected (Fig. 2a) following the methodology described in Ref. [1]. Then the acquired 3D point clouds were projected onto the camera focal plane (Fig. 2b), generating the RGB, range-corrected IR and depth projected images. These images were geometrically wrapped and registered (Fig. 2c) with RGB_hr_ so that different image modalities have pixel-wise correspondence. Finally, to reduce the number of fruits per image, and considering that fruit size is small compared with the image size, each capture was split into 9 images of 548 × 373 px (Fig. 2d).
